# Imaging and photodynamic therapy of prostate cancer using a theranostic PSMA-targeting ligand

**DOI:** 10.1007/s00259-023-06224-1

**Published:** 2023-04-15

**Authors:** Yvonne H. W. Derks, Melline G. M. Schilham, Mark Rijpkema, Esther M. M. Smeets, Helene I. V. Amatdjais-Groenen, Annemarie Kip, Sanne A. M. van Lith, Jill van de Kamp, J. P. Michiel Sedelaar, Diederik M. Somford, Michiel Simons, Peter Laverman, Martin Gotthardt, Dennis W. P. M. Löwik, Sandra Heskamp, Susanne Lütje

**Affiliations:** 1 Department of Medical Imaging, Nuclear Medicine, Radboud University Medical Center, Radboud Institute for Molecular Life Sciences, Geert Grooteplein Zuid 10, 6525GA Nijmegen, The Netherlands; 2 Department of Urology, Radboud University Medical Center, Nijmegen, The Netherlands; 3 Prosper Prostate Cancer Clinics, Nijmegen, The Netherlands; 4 Institute for Molecules and Materials, Systems Chemistry, Radboud University Nijmegen, Nijmegen, The Netherlands; 5 Department of Urology, Canisius Wilhelmina Hospital, Nijmegen, The Netherlands; 6 Department of Pathology, Radboud University Medical Center, Nijmegen, The Netherlands; 7 Department of Nuclear Medicine, University Hospital Aachen, Aachen, Germany

**Keywords:** Prostate-specific membrane antigen (PSMA), PSMA ligands, Prostate cancer, Photodynamic therapy (PDT), Theranostic agents, Intraoperative

## Abstract

**Purpose:**

Incomplete resection of prostate cancer (PCa) results in increased risk of disease recurrence. Combined fluorescence-guided surgery with tumor-targeted photodynamic therapy (tPDT) may help to achieve complete tumor eradication. We developed a prostate-specific membrane antigen (PSMA) ligand consisting of a DOTA chelator for ^111^In labeling and a fluorophore/photosensitizer IRDye700DX (PSMA-N064). We evaluated the efficacy of PSMA-tPDT using PSMA-N064 in cell viability assays, a mouse xenograft model and in an ex vivo incubation study on fresh human PCa tissue.

**Methods:**

In vitro, therapeutic efficacy of PSMA-N064 was evaluated using PSMA-positive LS174T cells and LS174T wild-type cells. In vivo, PSMA-N064-mediated tPDT was tested in immunodeficient BALB/c mice-bearing PSMA-positive LS174T xenografts. Tumor growth and survival were compared to control mice that received either NIR light or ligand injection only. Ex vivo tPDT efficacy was evaluated in excised fresh human PCa tissue incubated with PSMA-N064.

**Results:**

In vitro, tPDT led to a PSMA-specific light- and ligand dose-dependent loss in cell viability. In vivo, tPDT-induced tumor cell apoptosis, delayed tumor growth, and significantly improved survival (*p* = 0.004) of the treated PSMA-positive tumor-bearing mice compared with the controls. In fresh ex vivo human PCa tissue, apoptosis was significantly increased in PSMA-tPDT-treated samples compared to non-treated control samples (*p* = 0.037).

**Conclusion:**

This study showed the feasibility of PSMA-N064-mediated tPDT in cell assays, a xenograft model and excised fresh human PCa tissue. This paves the way to investigate the impact of in vivo PSMA-tPDT on surgical outcome in PCa patients.

**Supplementary Information:**

The online version contains supplementary material available at 10.1007/s00259-023-06224-1.

## Introduction

Prostate cancer (PCa) ranks as the second most common cancer and the fifth most frequent cause of cancer death among men worldwide [1]. At present, the curative treatment option for localized stages of PCa is radical prostatectomy, with or without pelvic lymph node dissection [2]. Unfortunately, the success rate of surgical PCa treatment is limited by two main factors. Firstly, wide local excision of malignant tissue is often difficult due to its close proximity to other vital anatomical structures in the lower pelvis, such as neurovascular bundles, the urinary bladder, pelvic floor musculature, or rectum wall. Hence, positive surgical margins occur in 5–30% of patients with organ-confined prostate cancer and 17–65% of patients with extraprostatic extension of the disease (pT3-pT4) [3, 4]. Presence and extent of positive surgical margins are associated with early disease recurrence and the application of adjuvant and/or salvage therapies [3–5]. Secondly, metastatic lymph nodes are difficult to detect intraoperatively and can be missed during pelvic lymph node dissection [6, 7]. Consequently, recurrence after prostatectomy is observed in 35% of patients [8, 9].

**Fig. 1:**
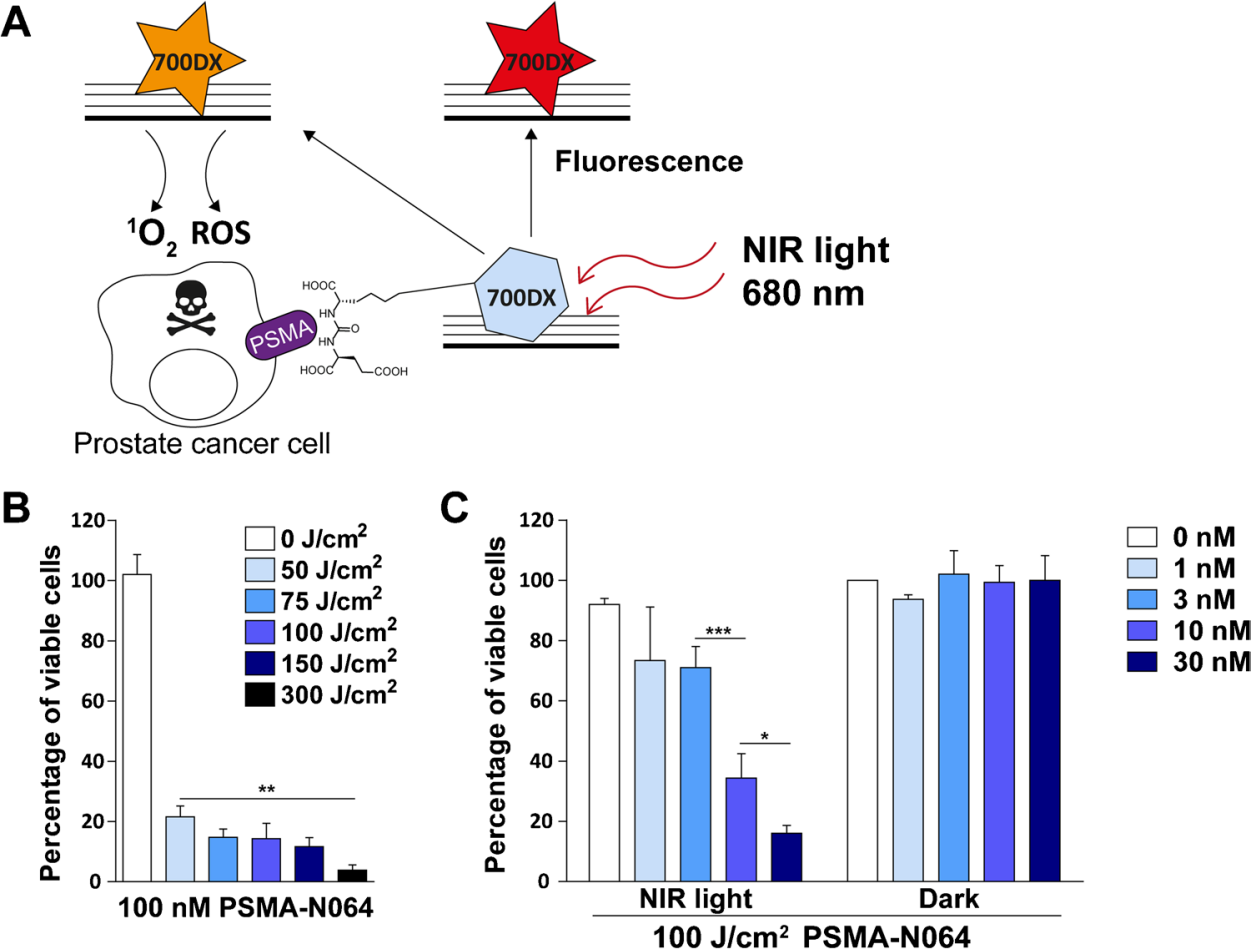
In vitro light and ligand dose optimization for tPDT with PSMA-N064**. A** Principle of PSMA-targeted IRDye700DX-mediated photodynamic therapy (PDT). **B** Cell viability of LS174T-PSMA tumor cells incubated with 100 nM PSMA-N064 and irradiated with 0, 50, 75, 100, 150, or 300 J/cm^2^ (450 mW/cm^2^). **C** Cell viability of LS174T-PSMA tumor cells incubated with 0, 1, 3, 10, or 30 nM PSMA-N064 after either a 100 J/cm^2^ radiant exposure (450 mW/cm^2^) or no light exposure (dark). ^*^p < 0.05; ^**^p < 0.01; ^***^p < 0.001. *NIR*, near-infrared; *PSMA*, prostate-specific membrane antigen; *ROS*, reactive oxygen species

Fluorescence image-guided surgery combined with photodynamic therapy (PDT) is a promising strategy to improve surgical treatment of PCa. PDT is a method to induce cell death by administration and activation of a photosensitizer. The photosensitizer is excited with a light source at a specific wavelength and releases light which can be used for intraoperative fluorescence imaging of PCa [10, 11]. Furthermore, the photosensitizer can produce singlet oxygen (^1^O_2_) and reactive oxygen species (ROS), highly toxic oxygen radicals (Fig. 1A) [12, 13]. In order to specifically treat PCa cells, photosensitizers can be coupled to prostate-specific membrane antigen (PSMA)-targeting ligands. PSMA is highly overexpressed in approximately 90% of localized PCa, metastatic lymph nodes, and distant metastases, with limited expression in healthy tissues [14, 15]. Combined with a light source focused on the tumor site, PSMA-targeted PDT (PSMA-tPDT) results in prostate tumor-specific cell killing with limited damage to surrounding tissues [16].

Previously, we synthesized a series of novel multimodal [^111^In]In-IRDye700DX-PSMA ligands and evaluated their intraoperative and multimodal prostate cancer imaging potential [17]. Here, we evaluated the potential of the lead candidate PSMA-N064 for a novel therapeutic approach: PSMA-targeted photodynamic therapy. PSMA-tPDT efficacy of PSMA-N064 was tested in cell culture experiments and a mouse xenograft model for proof-of-concept. For clinical translation, the therapeutic efficacy of the ligand was tested on fresh human PCa tissue samples.

## Materials and methods

### Synthesis of multimodal ligands

**Fig. 2:**
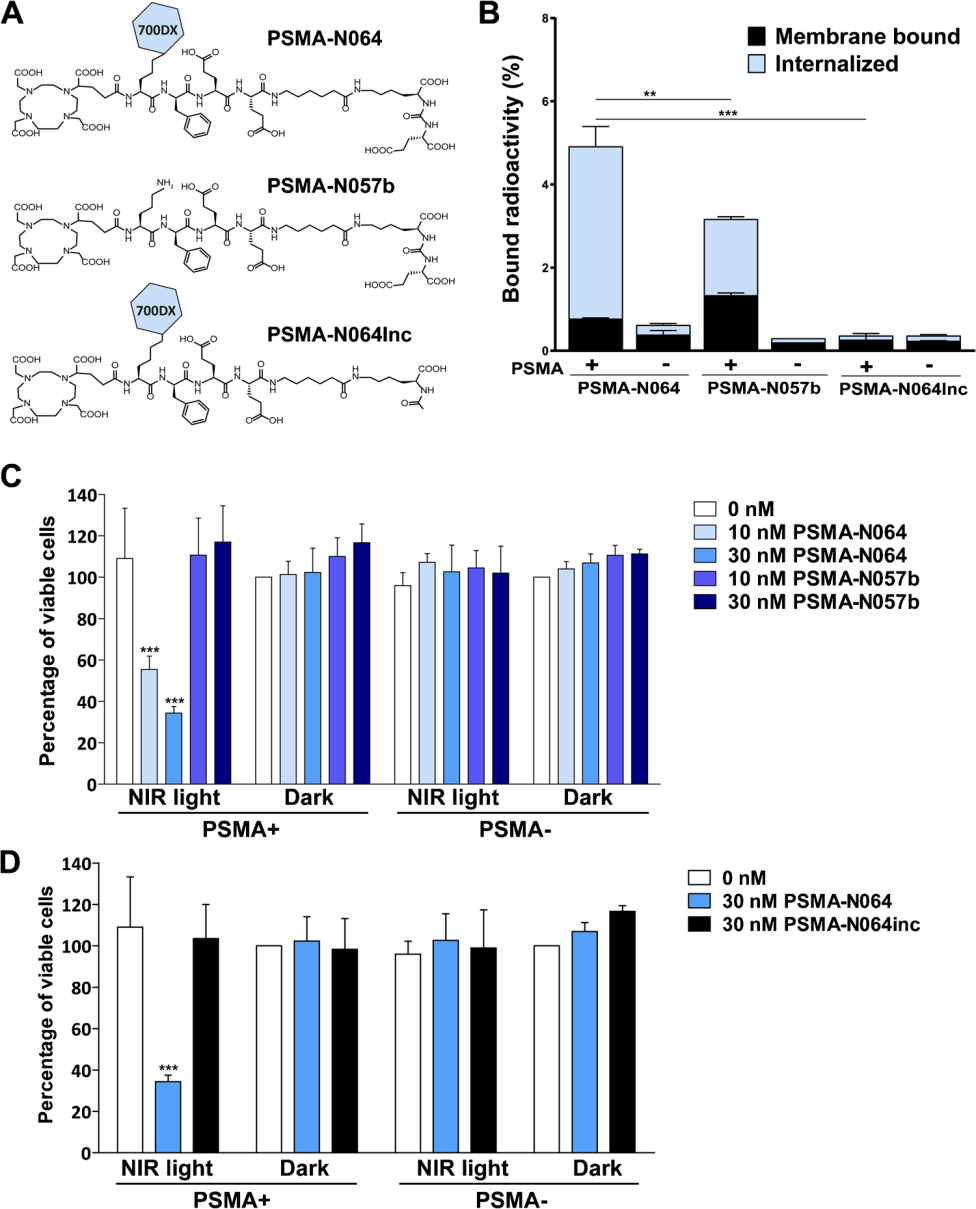
Specific uptake and PSMA-tPDT effects of PSMA-N064 in PSMA-positive cells. **A** Structures of PSMA-N064 and control ligands PSMA-N057b (no IRDye700DX) and PSMA-N064inc, lacking the glutamic acid in the PSMA-binding motif. **B** PSMA-receptor bound and internalized fraction of ^111^In-labeled PSMA-N064, PSMA-N057b, and PSMA-N064inc in LS174T PSMA-positive and negative cells***. C*** Cell viability of LS174T-PSMA (PSMA +) and LS174T wild-type (PSMA-) cells following incubation with 10 or 30 nM of PSMA-N064 or PSMA-N057b, after either a 100 J/cm^2^ radiant exposure (450 mW/cm^2^) or no light exposure (dark). **D** Cell viability of LS174T-PSMA (PSMA +) and LS174T wild-type (PSMA-) cells following incubation with 30 nM of PSMA-N064 or PSMA-N064inc, after either a 100 J/cm^2^ radiant exposure (450 mW/cm^2^) or no light exposure (dark). ^**^p < 0.01; ^***^p < 0.001*. NIR*, near-infrared; *PSMA*, prostate-specific membrane antigen

The glutamate-urea-lysine-based PSMA-targeting ligand PSMA-N064, containing IRDye700DX and 1,4,7,10-tetraazacyclododecane-1,4,7,10-tetraacetic acid (DOTA), was synthesized using a solid-phase chemistry. Two control ligands were synthesized, one that lacks the IRDye700DX fluorophore (PSMA-N057b) and one that lacks the glutamic acid in the PSMA-binding motif (referred to as PSMA-N064-incomplete (PSMA-N064inc, Fig. 2A)). A detailed description of the synthetic procedures and chemical analyses (HPLC, ESI-ion trap, MALDI-ToF) has been published previously [17].

### Cell culture

Cell lines were purchased from the American Type Culture Collection. Colon carcinoma cells (LS174T-WT), and LS174T colon carcinoma cells stably transfected with human PSMA using plasmid pcDNA3.1-hPSMA (LS174T-PSMA) were cultured in RPMI 1640 medium, supplemented with 10% fetal calf serum (Life technologies), and 2 mM glutamine (5% CO2, 37 °C). LS174T-PSMA cells were cultured in the presence of 0.3 mg/ml G418 geneticin [18].

### In vitrotPDT assays

LS174T-WT and LS174T-PSMA cells were cultured to confluency in 48-well plates. Cells were incubated for 2 h (5% CO_2_, 37 °C) with 0, 1, 3, 10, 30, or 100 nM of PSMA-N064, PSMA-N057b, or PSMA-N064inc in binding buffer (RPMI 1640 medium with 0.5% bovine serum albumin) in triplets. The triplicats were randomly distributed over the center of the plates considering the difference in light intensity within the NIR light-emitting diode (LED) [19]. As a negative control for NIR light irradiation effects, cells received phosphate-buffered saline (PBS) only (without addition of a PSMA ligand). As control for cellular toxicity of the PSMA ligands themselves, cells were incubated with PSMA ligand but not irradiated with NIR light. After incubation with PSMA ligands or PBS, cells were washed with PBS and a 0.5-ml fresh-binding buffer was added to each well. Subsequently, cells were irradiated with a NIR LED that emits light at a wavelength of 690 nm [19]. The cells were irradiated at NIR radiant exposures of 50, 75, 100, 150, or 300 J/cm^2^ (450 mW/cm^2^) and subsequently incubated for 1 h at 37 °C. Cytotoxic effects of PDT with PSMA ligands were determined with a CellTiter-GloTM assay (Promega Benelux) according to the manufacturer’s instructions. Binding buffer was replaced with a 100-µl fresh-binding buffer and 100 µl CellTiter-Glo® 2.0. Plates were shaken (2 min) and incubated for 10 min at room temperature (RT). To determine the metabolic activity of the cells in the form of adenosine triphosphate, the luminescence was measured in a Tecan Infinite® 200 PRO.

### Radiolabeling

PSMA ligands (1 µg/labeling; specific activity 5 MBq/µg) were radiolabeled with 5 MBq ^111^InCl_3_ (curium) in 0.5 M 2-(N-morpholino)ethanesulfonic acid buffer (twice volume of ^111^InCl_3_), pH 5.5, at 45 °C for 30 min under metal-free conditions [20]. After incubation, 50 mM ethylenediaminetetraacetic acid (EDTA) was added to a final concentration of 5 mM to chelate unincorporated ^111^InCl_3_. Labeling efficiency was determined by instant thin-layer chromatography (ITLC) using silica gel–coated paper (Agilent Technologies) and 0.1 M ammonium acetate containing 0.1 M EDTA, pH 5.5, as the mobile phase. To determine the effects of ^111^In labeling on tPDT efficacy, an in vitro tPDT assay was performed with and without radiolabeling of PSMA-N064 (3 and 30 nM, 5 MBq ^111^In).

### In vitro binding assay

The binding and internalization characteristics of [^111^In]In-DOTAGA-PSMA-N064 and [^111^In]In-DOTAGA-PSMA-N064inc (specific activity 5 MBq/µg) were compared using LS174T-PSMA and wild-type LS174T cells. 1.25 × 10^6^ cells/well were seeded and cultured to confluency in 6-wells plates followed by incubation at 37 °C for 2 h in a 1-ml binding buffer with 50,000 counts per minute (cpm) of ^111^In-labeled ligand (393 or 424 fmol/well for PSMA-N064 and PSMA-N064inc, respectively). Nonspecific binding was determined by coincubation with 2-(phosphonomethyl)pentane-1,5-dioic acid (2-PMPA, 21.57 μM). To retrieve the membrane-bound fraction, cells were incubated with acid buffer (0.1 M acetic acid, 154 mM NaCl, pH 2.6) for 10 min at 0 °C. Subsequently, cells were washed with PBS and lysed with 0.1 M NaOH. Membrane-bound and intercellular activity were measured in a gamma-counter (2480 WIZARD^2^, PerkinElmer) [18].

### Animal tumor model

All animal experiments were approved by the institutional Animal Welfare Committee of the Radboud University Medical Center and were conducted in accordance with the guidelines of the Revised Dutch Act on Animal Experimentation. Male BALB/c nude mice (Janvier, 8–10 weeks old) were housed in individually ventilated cages (Blue line IVC, 3–5 mice per cage), under standard nonsterile conditions with cage enrichment. There was free access to chlorophyll-free animal chow (Sniff Voer) and water. LS174T-PSMA cells (3 × 10^6^ cells in 100 µl RPMI 1640 medium) were subcutaneously inoculated into the right hind leg of the mice. The researchers were not blinded for the experimental groups and tumor-bearing mice were block-randomized based on tumor size.

### In vivo photodynamic therapy

Male BALB/c nude mice (*n* = 64) were s.c. inoculated with 3 × 10^6^ LS174T-PSMA cells and were included in the experiment once tumor size reached 50 mm^3^ (time to inclusion range 8–14 days). Groups 1 (PSMA-N064 + NIR) and 2 (PSMA-N064 without NIR) received 3 nM PSMA-N064 (200 µl/mouse) intravenously. Ligand dose was based on previous dose optimization studies of the PSMA-N064 ligand [17]. Groups 3 (PBS + NIR) and 4 (PBS without NIR) received PBS (200 µl/mouse) intravenously. After 2 h, mice in groups 1 and 3 were anesthetized (2.5% isoflurane inhalation anesthesia (Figure S1)), and tumors were irradiated with 150 J/cm^2^ NIR light for 10 min (300 mW/cm^2^) at a wavelength of 690 nm [19]. Based on the in vitro results and because of the single illumination protocol used, the higher light dose (150 J/cm^2^) was chosen. Six tumors per group were used for immunohistochemical analysis at 1 h (3 mice, γH2AX) or 24 h (3 mice, cleaved caspase-3) post tPDT. Furthermore, three mice from the treatment group (group 1, PSMA-N064 + NIR) and control group 3 (PBS + NIR) were used for photoacoustic imaging, immediately prior to irradiation, as well as 2 h and 24 h after irradiation. To monitor heating due to NIR light irradiation, the temperature of the area near the tumor and the total body temperature of the animals were measured during irradiation in four mice (rod thermometer: rectal and on covered skin of tumor area). After irradiation, all mice were placed tumor-side down on wet tissues to cool for 5 min. Analgesia (0.012 mg/ml buprenorphine, oral daily application) was applied in all groups one day prior until 3 days after NIR light exposure. Three times a week, mice were weighed and tumor diameter was measured in three dimensions with a caliper, by a biotechnician blinded for the experimental groups. When mice met one of the humane endpoints or a tumor volume above 1000 mm^3^ was measured, mice were euthanized by CO_2_/O_2_ asphyxiation. The following experiment-specific humane endpoints were used: tumor growth causing discomfort, severely reduced motility or signs of clinical discomfort (dehydration, 15% weight loss in less than 2 days). In the treatment group, (group 1, PSMA-N064 + NIR) two mice had to be sacrificed unrelated to tumor growth but due to the experiment-specific humane endpoint clinical discomfort. These mice were excluded from further analysis. From 5 mice per group, 0.25 ml of blood was collected from the facial vein by cheek puncture (day − 3 and day 6) and hemocytometry (leukocytes, hemoglobin, thrombocytes) was analyzed.

### In vivo fluorescence imaging and ultrasound-guided photoacoustic imaging

To measure bleaching (light-mediated destruction of photosensitizers) of IRDye700DX upon NIR light irradiation, immediately before and after tPDT, background subtracted fluorescence images were acquired of the anesthetized tumor-bearing mice with a fluorescence imaging system (Xenogen VivoVision IVIS Lumina II, Caliper Life Sciences). Near-infrared fluorescence (NIRF) images had an acquisition time of 10 s, excitation 640 nm, autofluorescence correction excitation 535 nm; both measured with the Cy5.5 filter and were analyzed using Living Image software version 4.2 (Caliper Life Sciences). Photoacoustic imaging is a technique that can provide a 3D-image of tumor StO_2_ by measuring oxygenated and deoxygenated hemoglobin. Photoacoustic imaging was performed (Vevo LAZR photoacoustic system, VisualSonics) prior to, as well as 2 and 12 h after irradiation with NIR light. During photoacoustic imaging, mice were anesthetized with 2.5% isoflurane inhalation anesthesia and kept warm with a heating pad. To facilitate acoustic contact between the transducer and the tumor, clear ultrasound gel was placed on the tumor. An emission wavelength of 850 nm was used. The pressure waves were detected by the transducer (MS550D: 22–55 MHz operating frequency MicroScan transducer, VisualSonics).

### Ex vivo tPDT human PCa samples

Seventeen patients who underwent radical prostatectomy with an ISUP-score of ≥ 2 based on pre-operative prostate biopsies were included. Directly after surgical resection of the prostate, fresh samples from the tumor (*n* = 4) and contralateral healthy region (*n* = 1) were taken from each patient using a biopsy gun (core length 17 mm). The location of the tumor was identified by visual inspection and palpation of the resected prostate. The ex vivo incubation protocol is shown in Figure S2, and was partly based on a previous published incubation study [17]. Four tumor samples from each patient were collected and randomly assigned to one of the following treatment groups: (1) treated (PSMA-N064 + NIR) tumor sample, (2) ligand-only tumor control, (3) NIR-only tumor control, (4) PBS-only tumor control. One healthy tissue sample taken from the contralateral prostate lobe from each patient was assigned to group 5, receiving the full treatment (PSMA-N064 + NIR). Based on a previous ex vivo imaging study [17] and a subsequent optimization study on samples of eight patients (data not shown), a 0.08 nmol/ml PSMA-N064 ligand in combination with 50 J/cm^2^ (300 mW/cm^2^) light dose were chosen. With these ligand and light doses, we saw the best balance between specific treatment effects and a specific damage due to NIR light alone. Samples were incubated for 4 h at 37 °C, 5% CO_2_ in 3 mL binding buffer (RPMI 1640 containing 0.1% w/v bovine serum albumin). For incubation, PSMA-N064 (0.08 nmol) was added to the buffers of three out of five samples: treated tumor group, treated healthy control group, and ligand-only tumor control group. After incubation, samples were washed with a 2.5-ml binding buffer followed by whole sample fluorescence imaging using a flatbed fluorescence scanner (Odyssey; channel, 700 nm; focus, 2.5 mm). Subsequently, samples in the treated tumor group, healthy control group, and irradiation-only tumor control group were irradiated with NIR light (50 J/cm^2^, 300 mW/cm^2^) using the LED. The study was performed in accordance with the Code of Conduct of the Federation of Medical Scientific Societies in the Netherlands and the 1964 Helsinki declaration and its later amendments or comparable ethical standards. The local institutional ethics committee of the Radboud University Medical Center approved this study (case number: 2019–5810). All samples and corresponding data were handled and stored anonymously.

### Immunohistochemistry

Treated and untreated LS174T-PSMA tumors were harvested at 24 h after NIR light irradiation (3 mice/treatment group) and fixated in 4% buffered formalin. Samples from the human tumor and benign region of the prostate were fixated in 4% buffered formalin 16 h after tPDT. Samples were embedded in paraffin and sectioned at 4 μm thickness. Tissue sections were stained with hematoxylin and eosin (H&E) for morphological assessment an immunohistochemically stained for PSMA (1:750 dilution, rabbit anti-PSMA, EPR6253, Abcam), cleaved caspase-3 (1:4000 dilution, rabbit anti human/mouse cleaved caspase-3, ASP175, Cell Signaling), and γH2AX (1:1000 dilution, rabbit anti-γH2AX, 20E3, Cell Signaling). Briefly, slides were deparaffinized by xylene wash and rehydrated using ethanol. For immunohistochemical staining, antigen retrieval was performed with 10 mM citrate pH 6.0 in PT-Module (10 min, 96 °C) and endogenous peroxidase activity was quenched with 3% H_2_O_2_ for 10 min. After a 30-min preincubation with 20% normal goat serum, slides were incubated for 60 min at RT with rabbit anti-PSMA (1:750), or in a humidified chamber at 4 °C overnight with rabbit anti-cleaved caspase-3 (1:4000) or rabbit anti-γH2AX (1:1000) antibodies. Next, slides were washed 3 times with 10 mM PBS and incubated with goat-anti-rabbit-biotin (1:200 Vector Laboratories) for 30 min at RT, followed by, Vectastain Elite ABC kit (Vector Laboratories) incubation for 30 min. After washing with PBS, diaminobenzine (DAB, Sigma-Aldrich) was used to visualize the bound secondary antibodies. All slides were counterstained with 3 times diluted hematoxylin (Klinipath) and mounted with a cover slip (Permount, Fisher Scientific).

### Quantitative analysis of apoptosis

The induction of apoptosis by PSMA-tPDT in PSMA-avid tumor regions in human PCa samples was quantified. Slide digitization was performed using a 3DHistech P1000 digital slide scanner (3DHistech, Budapest, Hungary) with a 20 × objective at resolution of 0.24 µm/pixel. In each tumor sample, multiple regions were annotated manually as PSMA-positive (2–4 regions) and PSMA-negative (2 regions) on the cleaved caspase-3 stained slides by cognitive comparison to PSMA-stained slides using the Automated Slide Analysis Platform (ASAP) software package. The cleaved capase-3-stained slides were analyzed using a previously developed algorithm, the automated color deconvolution [21]. With this algorithm, the caspase-3-staining staining was extracted from the background hematoxylin staining. This algorithm is extended by computing the ratio (positive pixels for a staining per region of interest) using automated Otsu thresholding on a resolution of 2 µm/pixel.

### Statistical analysis

Statistical analyses were performed with Graphpad Prism, version 5.03. Results are presented as mean ± SD. Differences in percentage cell viability in the in vitro studies were compared by the two-way ANOVA. Differences in tumor growth rate between the groups in vivo were compared with the one-way ANOVA and Bonferroni’s posttests. Survival between the groups was compared with the log-rank (Mantel-Cox) test. Differences in the ratio of cleaved caspase-3 positive pixels in PSMA-positive and PSMA-negative regions in the ex vivo human PCa samples were assessed using unpaired Student’s *t*-test. A *p*-value below 0.05, two sided, was considered significant.

## Results

### tPDT with PSMA-N064 induces dose-dependent cell death

Compared with PSMA-positive cells that did not receive any treatment, cell viability decreased to 21% ± 3.6% upon irradiation with the lowest light dose (50 J/cm^2^), and further decreased in a light dose-dependent manner, down to a cell viability of 4% ± 1.7% when irradiated with 300 J/cm^2^ (Fig. 1B). A significant difference in cell viability was observed between the cells treated with 50 and 300 J/cm^2^ (*p* = 0.002). In a subsequent study, LS174T-PSMA cells were incubated with 0, 1, 3, 10, or 30 nM PSMA-N064 and irradiated with NIR light (100 J/cm^2^). Compared with PSMA-positive cells that did not receive any treatment (92% ± 2%), cell viability after tPDT with 1 nM PSMA-N064 was 73% ± 17.8%, and decreased in a dose-dependent manner down to 16% ± 2.6% when incubated with 30 nM PSMA-N064. A significant drop in viability was observed in the 3 versus 10 nM groups (*p* = 0.0002) and the 10 versus 30 nM groups (*p* = 0.019). No significant loss in cell viability was observed upon incubation of ligand without irradiation (Fig. 1C).

### PSMA-N064 shows specific uptake and tPDT effects in PSMA-expressing tumor cells

Next, PSMA-specific binding and tPDT effects were compared between PSMA-N064 and the control ligands PSMA-N057b (no IRDye700DX) and PSMA-N064inc (incomplete PSMA-binding motif, Fig. 2A). We verified the PSMA-binding potential of PSMA-N064 in a binding and internalization assay using LS174T PSMA-positive and negative cells (Fig. 2B). Incubation with ^111^In-PSMA-N064 revealed a membrane-bound and internalized fraction of 0.8% ± 0.02% and 4.1% ± 0.48%, respectively. In comparison, PSMA-N057b showed a membrane-bound and internalized fraction of 1.3% ± 0.06% and 1.8% ± 0.06%, respectively. Furthermore, no specific binding and internalization upon incubation with control ligand ^111^In-PSMA-N064inc was observed, demonstrating the requirement of an intact PSMA-binding motif for binding to PSMA-positive cells.

Next, the PSMA-specific tPDT effects of PSMA-N064 were examined and compared to its two controls in vitro. Upon incubation with 10 nM or 30 nM PSMA-N064, LS174T-PSMA cell viability was 56% ± 6.4% and 34% ± 3.2%, respectively. During incubation with PSMA-N057b or PSMA-N064in, no loss in cell viability was observed (*p* < 0.001, Fig. 2C and D). All controls, consisting of irradiated PSMA-negative LS174T-WT cells and non-irradiated LS174T-PSMA and LS174T-WT cells, show a cell viability around 100% (Fig. 2C and D). Last, the effect of ^111^In radiolabeling on IRDye700DX stability and thus tPDT efficacy was determined. LS174T-PSMA cell viability after tPDT did not significantly differ between ^111^In-labeled PSMA-N064 (2 h) and unlabeled PSMA-N064 (*p* = 0.16, Figure S3).

### PDT causes photobleaching of PSMA-N064 and decreases oxygenation in treated tumors

The potential of PSMA-N064 for tPDT was determined in vivo in LS174T-PSMA tumor–bearing mice. At the day of irradiation, mean tumor sizes were 94.4 ± 42.5 mm^3^ (group 1: PSMA-N064 + NIR), 93.5 ± 36.3 mm^3^ (group 2: PSMA-N064 without NIR), 100.8 ± 46.8 mm^3^ (group 3: PBS + NIR), and 98.7 ± 39.7 mm^3^ (group 4: PBS without NIR), which did not significantly differ between groups (*p* = 0.96). tPDT did not lead to hematotoxicity (Figure S4). During irradiation, an increase in temperature was observed (whole body: from 31.4 ± 0.7 °C to 39.0 ± 1.0 °C, tumor area: 31.5 ± 0.9 °C to 43.1 ± 2.6 °C, Figure S5).

**Fig. 3:**
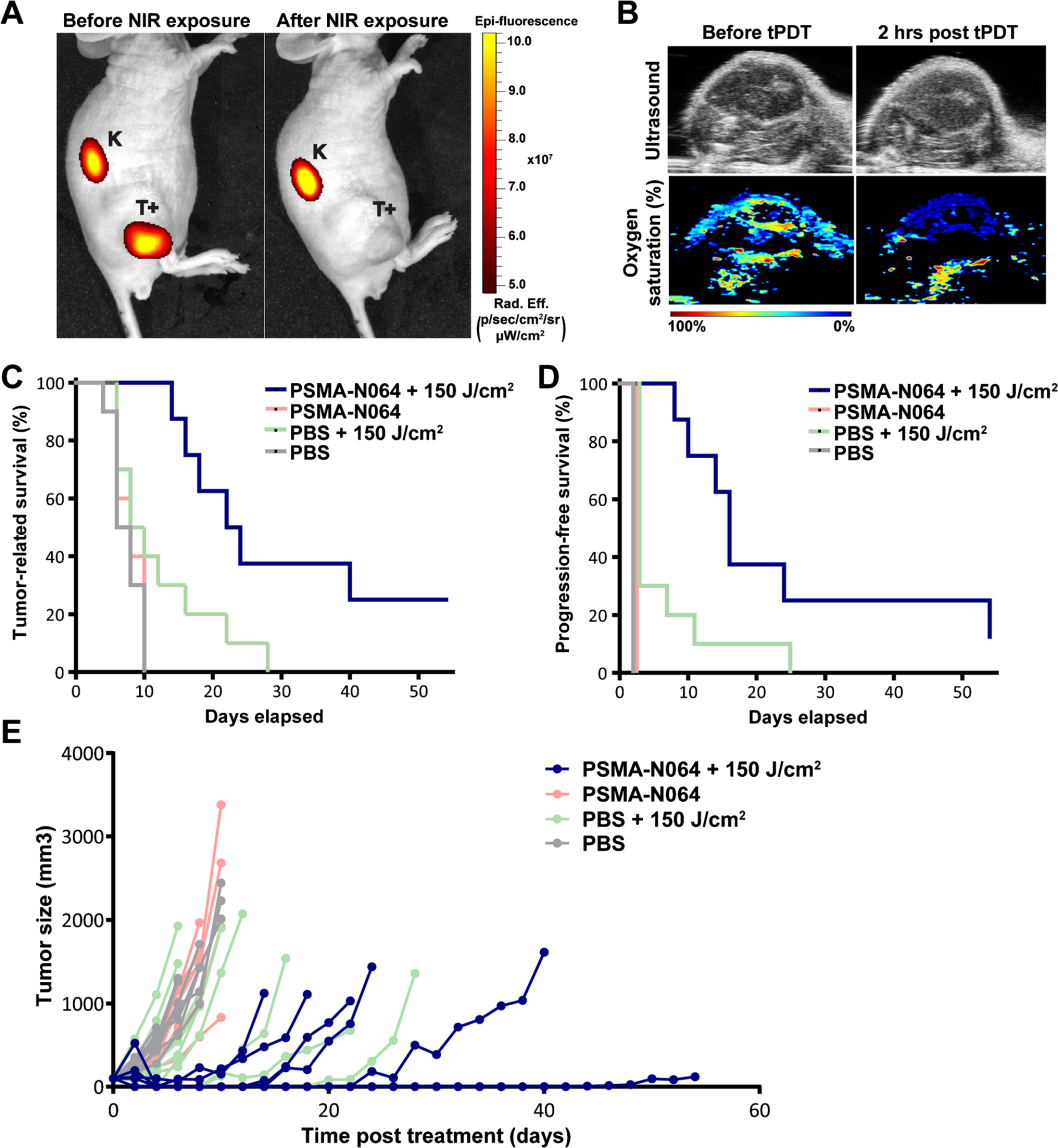
PSMA-tPDT using PSMA-N064 inhibits tumor growth and significantly improves survival.** A** Photobleaching of PSMA-N064 upon NIR light exposure. NIRF images of mice with s.c. LS174T-PSMA (T +) tumors after i.v. injection of PSMA-N064 (3 nmol) before (left) and directly after (right) tPDT (2 h p.i.). Ligands are excreted via the kidneys (K). **B** Oxygen saturation of LS174T-PSMA tumor before and 2 h after tPDT, measured via photoacoustic imaging. **C, D** Kaplan–Meier plots of overall and progression-free survival and **E** tumor growth in male BALB/c nude mice (10 mice/group) with s.c. LS174T-PSMA tumors after i.v. injection of 3 nmol PSMA-N064 or PBS (control), followed by NIR light exposure of 150 J/cm^2^ (2 h p.i., 300 mW/cm^2^) or no exposure (dark, control). Each line represents one mouse. *NIR*, near-infrared; *PBS*, phosphate-buffered saline; *PSMA*, prostate-specific membrane antigen; *tPDT*, targeted photodynamic therapy

Before and after NIR exposure, mice from group 1 were imaged with a fluorescence camera to evaluate tumor accumulation of the ligand and to monitor photobleaching of PSMA-N064 [12]. Specific accumulation of PSMA-N064 was observed in all PSMA-positive tumors. Loss of the IRDye700DX fluorescent signal was seen in the tumor region after irradiation with NIR light. As expected, no bleaching was observed in areas that were not exposed to light (e.g., kidneys) (Fig. 3A).

Tumor oxygenation status was determined before, as well as 2 and 12 h after tPDT. StO_2_ levels decreased from 43.8% ± 6.3% before treatment to 14% ± 3.6% and 16% ± 4.4% at 2 and 12 h post tPDT for PSMA-N064 + NIR-treated mice respectively (Figure S6 and representative images in Fig. 3B). In control mice receiving PBS + NIR light, a decrease in StO_2_ levels from 36.7% ± 8.6 to 21.7% ± 3.8% at 2 h and 21.3% ± 7.0% at 12 h after treatment was observed (Figure S6).

### tPDT using PSMA-N064 inhibits tumor growth and significantly improves survival

To assess the tPDT efficacy of PSMA-N064, tumor growth and survival after tPDT were monitored in all groups of mice. Analysis of tumor-related survival (until tumors reached a size of 1000 mm^3^) revealed a median survival of 23 days in treated mice (PSMA-N064 + NIR, group 1), which was significantly prolonged compared with mice in the ligand-only control group (group 2, 8 days), mice in the irradiation-only control group (group 3, 9 days), or mice that received neither ligand nor NIR light irradiation (group 4, 7 days, *p* = 0.004) (Fig. 3C). In addition, progression-free survival, defined as time until measurable recurrence, was prolonged in treated mice (median progression-free survival of 16 days) as compared to the control groups (median progression-free survival of 2 days for groups 2–4) (*p* = 0.052) (Fig. 3D). The tumor growth of individual treated mice and mice in control groups is depicted in Fig. 3E. Figure S7 depicts the mean relative tumor growth for all treatment groups and the percentage tumor growth inhibition (%TGI) 10 days after PDT and at the day of sacrifice. At the end of the experiment, 55 days after PSMA-targeted PDT, fluorescence imaging revealed that one treated mouse showed a small tumor nodule. In the other mouse, no fluorescence signal could be detected (Figure S8A). However, PSMA-based immunohistochemical assessment of the tumor site revealed the presence of a tiny tumor nodule in both mice (Figure S8B).

### tPDT using PSMA-N064 increases apoptosis in LS174T-PSMA tumors

**Fig. 4:**
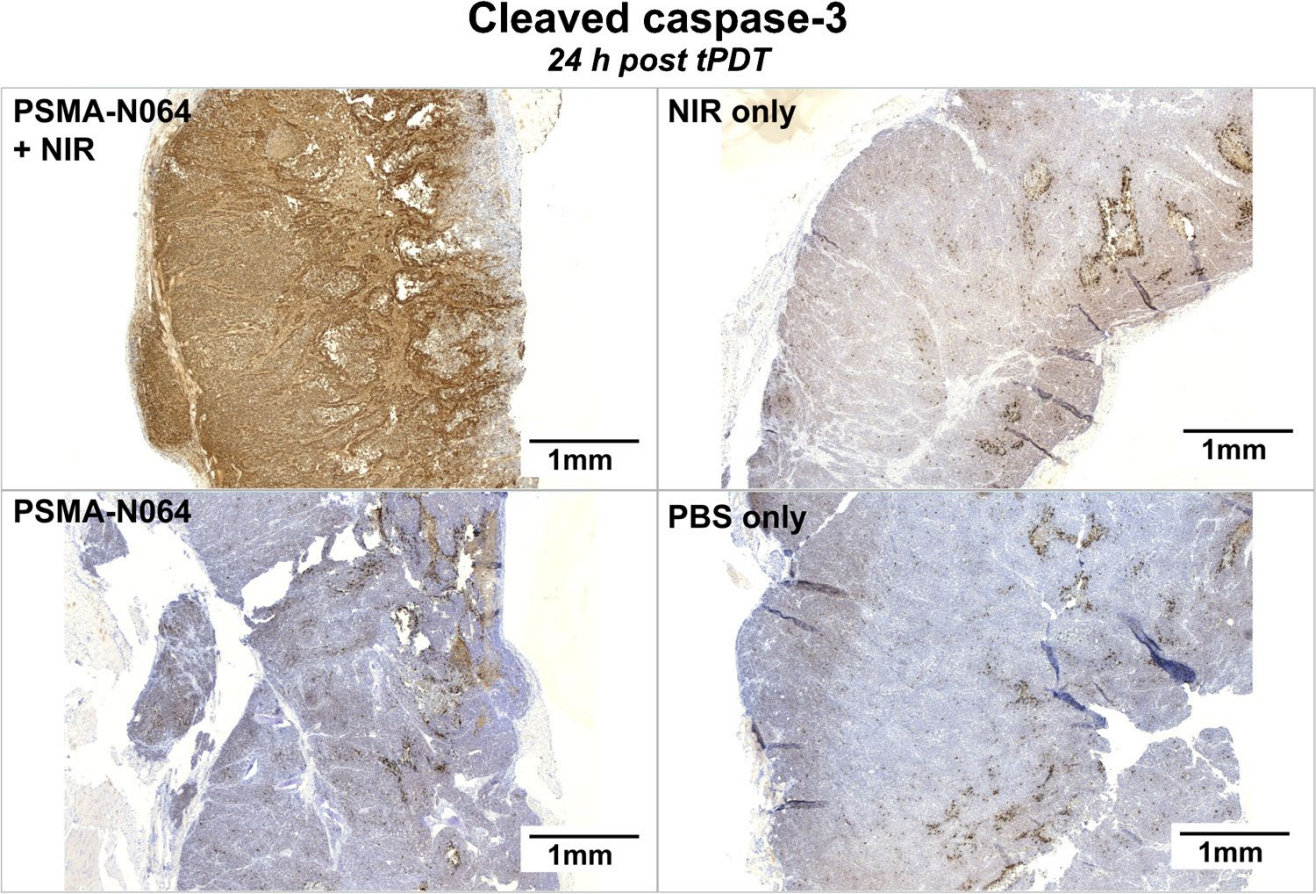
Increase of apoptosis in LS174T-PSMA tumors after tPDT using PSMA-N064. Cleaved caspase-3 staining of subcutaneous LS174T-PSMA tumors after intravenous injection of PBS or 3 nmol of PSMA-N064, followed by NIR light exposure of 150 J/cm^2^ (300 mW/cm^2^) 2 h after injection or no exposure. Tumors were dissected 24 h post tPDT. *NIR*, near-infrared; *PBS*, phosphate-buffered saline; *PSMA*, prostate-specific membrane antigen; *tPDT*, targeted photodynamic therapy

Visual assessment of cleaved caspase-3 staining in fully-treated LS174T-PSMA tumors (group 1) showed a large increase in apoptotic cells, which was homogenous throughout the tumor, compared with the control tumors groups (groups 2–4) (Fig. 4). However, mixed results were observed in group 3 (only NIR exposure), where one third of the tumors showed more cleaved caspase-3 positive cells in the rim of the tumor near the skin (Figure S9).

### PSMA-N064 accumulates in human PSMA-positive tissue and induces apoptosis upon tPDT

**Fig. 5:**
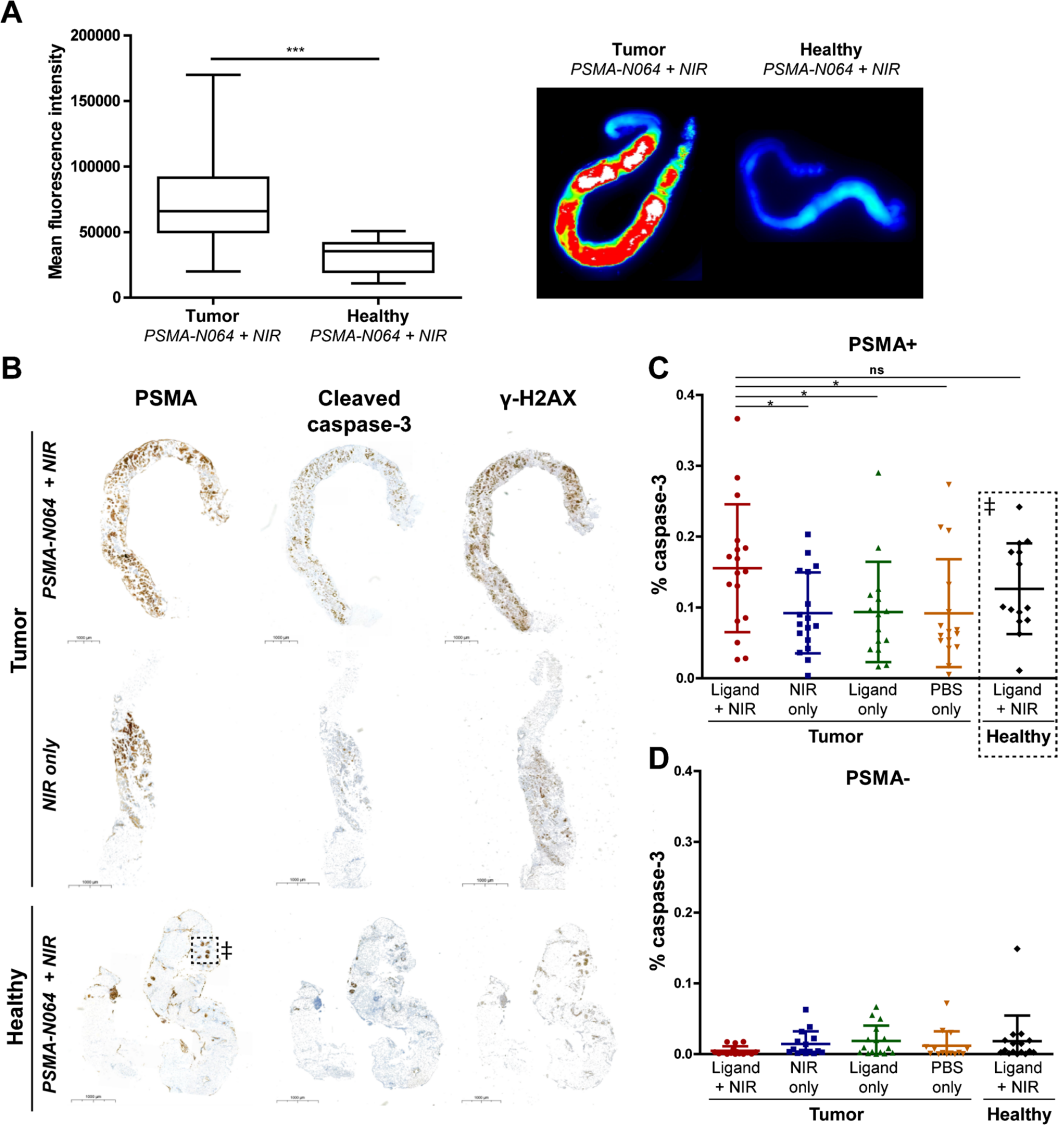
Ex vivo PSMA-tPDT on human PCa samples.** A** Fluorescence quantification and representative macroscopic fluorescence images of PSMA-N064 incubated (0.08 nmol) tumor samples and contralateral healthy control samples. **B** IHC staining of cleaved caspase-3 and γ-H2AX shows co-localization with PSMA-positive regions in a representative patient sample. A double dagger (^‡^) indicates dotted box: microscopic PSMA-positive regions were observed in healthy tissue sample. **C** Quantitative analysis of cleaved caspase-3 IHC staining in PSMA-positive regions. A double dagger (^‡^) dotted box: PSMA-positive areas in healthy control samples. **D** Quantitative analysis of cleaved caspase-3 IHC staining in PSMA-positive regions. Quantification is presented as percentage of tumor region expressing caspase-3 (*y*-axis).^*^p < 0.05; ^***^p < 0.001; ns, not significant; *NIR*, near-infrared; *PBS*, phosphate-buffered saline; *PSMA*, prostate-specific membrane antigen

Fluorescence flatbed scanning showed increased accumulation of the PSMA-N064 in human PCa tissues (mean fluorescence intensity (MFI) 76,578 ± 41,939) compared with tissue from the healthy prostate region (contralateral) (MFI 31,897 ± 12,609, *p* < 0.05), indicating specific uptake of the ligand in PSMA-expressing tumor tissue (Fig. 5A). An example of the fluorescence imaging is shown in Fig. 5A.

To study the effect of tPDT on human PCa and healthy prostate tissue, DNA double-strand breaks (γH2AX) and apoptosis (cleaved caspase-3) were analyzed immunohistochemically. An increase in cleaved caspase-3 and yH2AX staining was observed upon visual analyses of the PSMA-positive regions of the treated tumor samples (PSMA-N064 + NIR) compared with PSMA-positive regions in the tumor control groups, indicating an increase in apoptosis and double-strand DNA damage upon PSMA-tPDT. Representative images of the PSMA, cleaved caspase-3 and γH2AX staining in a treated tumor sample, NIR-only tumor control sample and treated healthy control sample are depicted in Fig. 5B. In PSMA-positive tumor regions, quantitative analysis showed an increased percentage of cleaved caspase-3 positive pixels in treated samples (0.16 ± 0.09) compared to NIR-only (0.09 ± 0.06, *p* = 0.021), ligand-only (0.09 ± 0.07, *p* = 0.041), and PBS-only samples (0.09 ± 0.08, *p* = 0.037) (Fig. 5C). Even in the healthy control samples, microscopic PSMA-positive areas were observed (e.g*.*, area indicated by the dotted box in Fig. 5B), these PSMA-positive areas also showed an effect of tPDT (Fig. 5C). Importantly, within the treated tumor samples and these healthy control samples, the ratio of cleaved caspase-3 positive pixels in PSMA-positive regions was significantly higher compared with the PSMA-negative regions, indicating the PSMA-specificity of the tPDT treatment (*p* < 0.05) (Fig. 5C-D).

## Discussion

To prevent recurrences after PCa surgery, we developed a theranostic PSMA ligand called PSMA-N064, which consists of a PSMA-binding motif, the fluorophore/photosensitizer IRDye700DX, as well as a chelator for ^111^In-labeling. Together with the findings from previous studies, these results suggest that multiple applications can be achieved with this multimodal ligand, including surgical guidance toward metastatic lymph nodes via gamma probe detection of ^111^In and real-time intraoperative fluorescence imaging to visualize and delineate the primary tumor [17]. Importantly, these ligands enable end-of-surgery PSMA-tPDT to destruct (invisible) tumor remnants that were deemed unresectable. Previously, we assessed the intraoperative imaging potential of PSMA-N064 [17], while in this study we set out to assess the therapeutic potential of PSMA-N064 for the innovative PSMA-tPDT approach. Withal, potent tPDT effects were observed both in vitro and in vivo, leading to tumor growth inhibition and prolonged survival of mice. Moreover, as a relevant step towards clinical translation, the PSMA-tPDT potential was assessed in fresh PCa tissue from the intended patient population, showing increased cell death in PSMA-positive regions.

The results of our in vitro studies showed a light and ligand dose-dependent decrease in cell viability of PSMA-expressing cells treated with tPDT, whereas the tumor cells remained viable in all control conditions. These results are in line with the study of Chen et al. (2017), who found a 99.8% loss in cell viability after incubation with 100 nM PSMA ligand YC-9, containing the IRDye700DX [22]. Elaborating on this research, in our study two control ligands PSMA-N057b (no dye) and PSMA-N064inc (no PSMA-binding motif) were included. Incubation with the control ligands did not cause any tPDT-induced cell death, showing that besides the presence of the photosensitizer, epitope binding is necessary for effective PSMA-tPDT. The radio bleaching effect of ^111^In labeling of PSMA-N064 on tPDT efficacy was evaluated and results show no difference in tPDT effect when PSMA-N064 was labeled with ^111^In (Figure S3). This is important considering the potential clinical application of the multimodal ligand for intraoperative gamma probe detection, (fluorescent) visualization of PSMA-expressing tumor lesions, and subsequent tPDT.

In our in vivo study, we observed an inhibition of tumor growth and a significant survival benefit in the tPDT-treated group compared to the controls. Yet, some effects (e.g., apoptosis induction and lower oxygenation in the tumor) were also observed in the NIR-only control groups, presumably due to heating of the tumor tissue, which might be prevented if lower light dose rates are used, as described by Okuyama et al. [23]. In a clinical setting, lasers will be applied instead of a LED device, which are more precise and cause less tissue heating [24].

Similar in vivo tPDT effects were observed by Lütje et al. using an anti-PSMA D2B antibody conjugated with IRDye700DX [11]. In this study, 80 µg of D2B conjugated with IRDye700DX was injected in mice prior to PDT experiments, with an absolute uptake of 1.2 nmol/g in the LS174T PSMA-positive tumor. In comparison, the 3 nmol injection of PSMA-N064 in our experiments led to an almost ten times lower absolute uptake of 0.14 nmol/g [17]. Logically, achieving high tumor uptake is preferable in order to acquire clear fluorescence signals and produce the maximum amount of oxygen radicals (PDT effects). Nonetheless, overall small molecule PSMA ligands, such as PSMA-N064 extravasate quickly, have a better tumor penetration, clear more rapidly from the blood, and show higher tumor-to-background ratios compared to antibodies [15, 25]. Since a higher tumor-to-background ratio may contribute to improved tumor margin assessment and rapid tumor-targeting enables tracer injection at the day of surgery, which is often preferred in clinical practice, small molecule PSMA ligands are preferred over antibodies [10, 26].

Three preclinical studies already demonstrated the feasibility of PSMA-tPDT using IRDye700DX-based low molecular weight PSMA ligands. Wang et al. showed selective and specific tumor uptake of the PSMA-1-IR700 ligand, leading to an effective inhibition of PSMA-positive PC3-PIP tumor progression [27]. Preclinical tPDT using the PSMA ligand YC-9 by Chen et al. resulted in significant tumor growth delay and increased the median survival of the PC3-PIP tumor mice compared to control groups, including untreated, light alone, and YC-9-alone groups [22]. However, in these studies, multiple treatment cycles of ligand injection and light exposure where applied. For clinical application, we envision illumination will be performed only once during surgery, directly after tumor resection. The single illumination protocol performed in the current study, therefore better resembles the clinical situation for intraoperative use. Recently, Capozza et al. did a full characterization of their 700DX-conjugated PSMA ligand in vitro and in vivo in different prostate cancer cell lines confirming the efficacy of PSMA-tPDT [28].

Yet, all of the abovementioned studies are performed in cell lines and s.c. tumor models that do not adequately reflect the heterogenous prostate cancer patient population. Therefore, the current in vitro and in vivo results in the LS174T-PSMA cell line are primarily a first proof-of-concept of PSMA-N064-mediated tPDT. Nonetheless, a previous direct comparison of the LNCaP and LS174T-PSMA xenograft models did not show major differences in ^111^In-PSMA-I&T tracer uptake between these models [29], suggesting that PDT effects of PSMA-N064 can be evaluated in the transfected LS174T-PSMA.

The first multimodal PSMA tracer for PCa detection, resection, and subsequent tPDT was developed by Harmatys et al. called LC-pyro [30]. With this porphyrin photosensitizer-based PSMA ligand fluorescence imaging and ^64^Cu chelation for PET/CT imaging is feasible. First results showed a high tumor accumulation and potent tPDT effects in a mice xenograft model. However, the IRDye700DX used in the current study has an excitation range within the NIR spectrum (700 nm), leading to a deeper penetration of light in tissue when compared to non-NIR photosensitizers such as porphyrins [31]. Moreover, IRDye700DX is highly photostable [16, 32]. Therefore, use of IRDye700DX is preferred for tPDT applications.

Recent literature shows that PDT does not only induce direct tumor cell killing, but also induces systemic anti-tumor immunity as a response to signals excreted by necrotic and apoptotic cells [33, 34]. This eventually may lead to pro-inflammatory anti-tumor activity, accompanied by immune memory [35, 36]. However, due to the use of human PSMA-positive LS174T tumors in immune deficient mice, or the use human tissue in an ex vivo setting, we were not able to study the effects of tPDT on the immune response in the current study.

As preclinical models often use s.c. human tumors with a homogenous target expression, accurate translatable information on specific tumor accumulation, and contrast between tumor and healthy adjacent tissue is not provided. Furthermore, variation in target antigen expression, and thus treatment effects within a patient population is not adequately reflected. Hence, for clinical translatability, we assessed the therapeutic potential of PSMA-N064 in fresh human PCa tissue samples [17, 37, 38]. This allowed us to test the specific tumor accumulation of PSMA-N064 and its tPDT effects on human PCa and normal tissue from the intended patient population, with a heterogenous physiological PSMA expression. In 17 patients, the ex vivo incubation experiment showed that tPDT with PSMA-N064 results in apoptosis in the PSMA-expressing areas (Fig. 5C–D). Within these tissue samples, the amount of apoptosis in PSMA-positive regions was significantly higher compared with the PSMA-negative regions, demonstrating the PSMA-specificity of the tPDT treatment. Nonetheless, we observed increased apoptosis and DNA damage in the PSMA-positive regions of all treatment groups, compared with their corresponding PSMA-negative regions. A possible explanation could be that tumor cells are more susceptible to external effects (e.g., heating, experimental procedures) compared to healthy prostate cells. However, this hypothesis needs further examination. A limitation of the ex vivo incubation protocol is that there is no circulation in excised tissue samples, and tracer uptake occurs through passive diffusion which does not represent the normal vascular delivery route of the tracer in patients.

In conclusion, the results obtained in this study showed the feasibility of PSMA-N064 ligand-induced PSMA-tPDT. In vitro tPDT led to PSMA-specific light dose and ligand dose-dependent cell death. In vivo tPDT significantly delayed tumor growth and improved survival of tumor-bearing mice. Furthermore, freshly excised human PCa tissue showed increased apoptosis in PSMA-positive regions following ex vivo PSMA-tPDT. Hence, this is the first study to demonstrate the tPDT potential of PSMA ligands on patient samples, bridging the gap towards clinical use of this new theranostic application. In the future, PSMA-tPDT might improve the outcomes of PCa surgery by means of intraoperative fluorescence imaging and tPDT of any remaining tumor cells.

## Supplementary Information

Below is the link to the electronic supplementary material.

Supplementary file1 (PDF 829 KB)

## Data Availability

The datasets generated during and/or analyzed during the current study are available from the corresponding author on reasonable request.

## Abbreviations

NIRF: Near-infrared fluorescence imaging
PCa: Prostate cancer
PSMA: Prostate-specific membrane antigen
tPDT: Targeted photodynamic therapy
