# Supplementary material for: Imaging and photodynamic therapy of prostate cancer using a theranostic PSMA-targeting ligand
Source: Eur J Nucl Med Mol Imaging. 2023 Apr 15;50(9):2872–84. doi: 10.1007/s00259-023-06224-1 (PMC10317872; doi:10.1007/s00259-023-06224-1)
Supplement: Supplementary file 1 — Supplementary file1 (PDF 829 KB) [file 259_2023_6224_MOESM1_ESM.pdf]

Supplementary Results

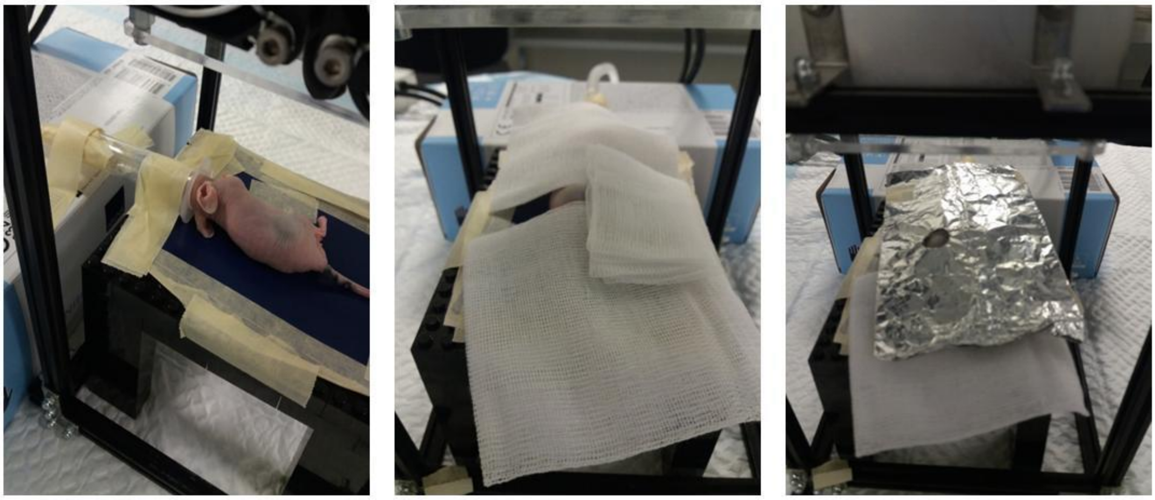

Figure S1. tPDT in vivo setup. Male BALB/c nude mice are irradiated with 150 J/cm^2 (300 mW/cm^2) with a near infrared (NIR) light-emitting diode (LED). During irradiation, mice were given 2.5% isoflurane inhalation anesthesia via a tube. Moreover, mice are covered with compresses and aluminum foil to protect from the NIR light and heat, except the LS174T-PSMA subcutaneous tumor which was exposed to the NIR light.

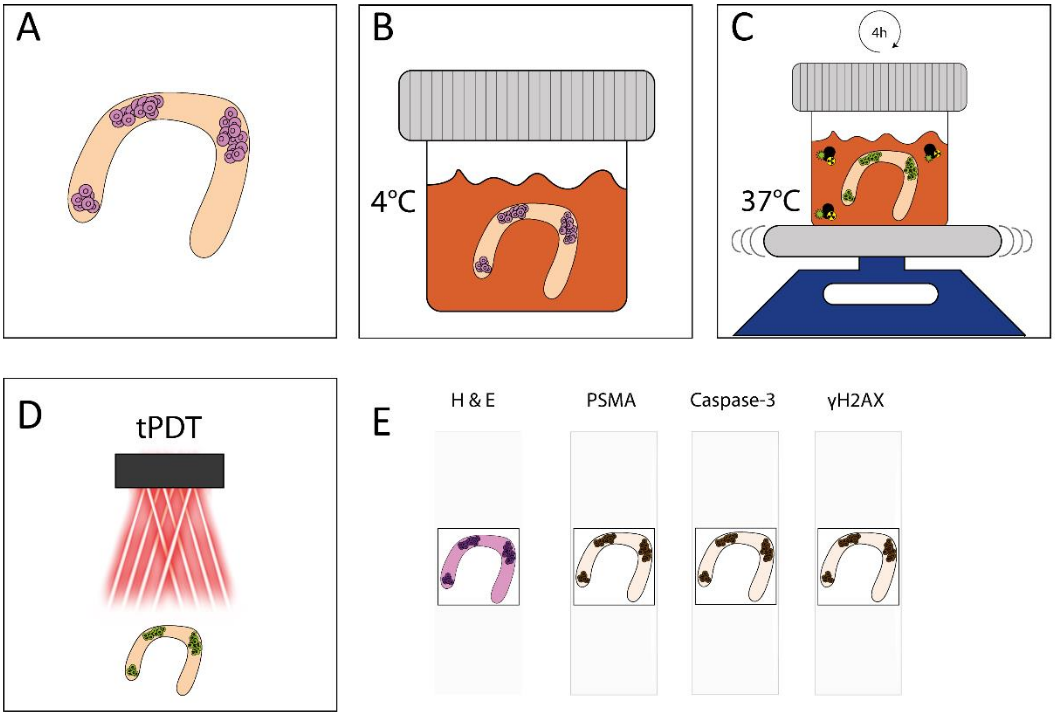

Figure S2. Protocol *ex vivo* incubation study human PCa samples. (A) Directly after surgical resection, fresh samples from the tumor (n=4) and contralateral healthy region (n=1) were taken from the prostate using a biopsy gun. The location of the tumor was identified by visual inspection and palpation of the resected prostate. **(B)** Samples were placed in cold binding buffer (RPMI 1640 containing 0.1% w/v bovine serum albumin). **(C)** Samples were incubated for four hours at 37 °C, 5% CO_2 in 3 mL binding buffer. For each patient, 0.05 nmol of PSMA-N064 was added to the buffers of three out of five samples: fully-treated tumor

sample group, contralateral-(healthy) control group and ligand-only tumor-control group. After incubation, the samples were washed with 2.5 ml binding buffer. **(D)** Prostate samples in the fully-treated tumor sample group, healthy-control group and NIR-only tumor group were irradiated with NIR light (50 J/cm^2, 300 mW/cm^2). **(E)** Samples were fixated in 4% buffered formalin 16 h after tPDT. Tissues were subsequently embedded in paraffin and sectioned at 4 µm thickness. Tissue sections were stained with hematoxylin and eosin (H&E) to visualize morphology. Tissue sections were immunohistochemically stained for PSMA, cleaved caspase-3 or γH2AX.
NIR= near infrared; *PCa*= prostate cancer; *PSMA*= prostate-specific membrane antigen; *tPDT*= targeted photodynamic therapy.

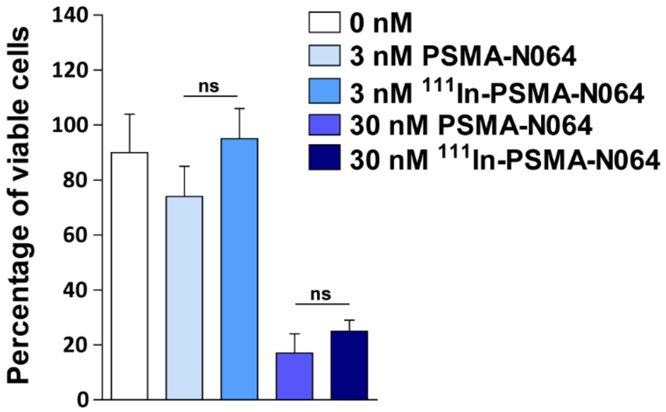

Figure S3. The effect of ^{111}In radiolabeling on tPDT efficacy. Cell viability of LS174T-PSMA cells following incubation with 3 or 30 nM ^{111}In-PSMA-N064 or unlabeled PSMA-N064 after a 100 J/cm^2 NIR radiant exposure (450 mW/cm2).
ns= not significant; *NIR*= near infrared; *PSMA*= prostate-specific membrane antigen; *tPDT*= targeted photodynamic therapy.

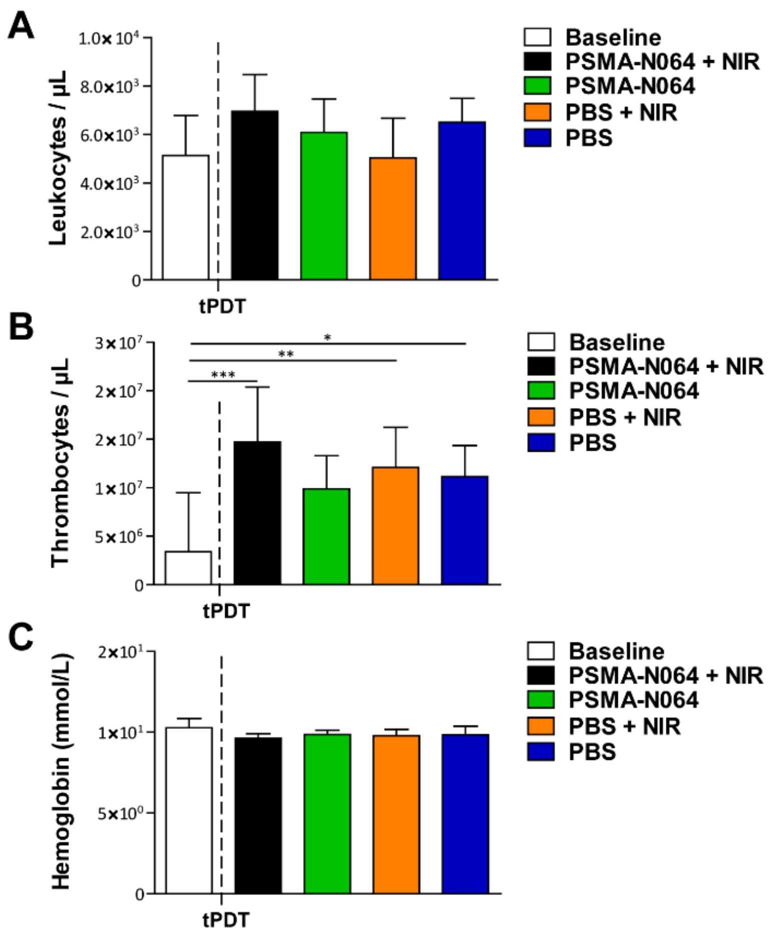

Figure S4. Hematoxicity measurements before and after tPDT treatment.
Hematotoxicity of tPDT was examined in male BALB/c nude mice with s.c. LS174T-PSMA tumors. The bars depicted in white represent the baseline before treatment on day -3. Mice received 150 J/cm^2 near infrared light irradiation. **(A)** Hemocytometry visualized for leukocytes (amount of cells present/µL) show no significant differences between before and after tPDT treatment or between groups. **(B)** Hemocytometry visualized for thrombocytes (amount of cells present/µL) show significant differences between baseline and after treatment for group 1 (PSMA-N064) ($t=4.757$, $P<0.05$), group 3 (PBS + NIR) ($t=3.958$, $P<0.05$), and group 4 (PBS) ($t=3.451$, $P<0.05$). However, no significant differences were seen between groups after treatment. **(C)** Hemocytometry visualized for hemoglobin (mmol/L) show no significant differences between before and after tPDT treatment or between groups.
NIR= near infrared; *PBS*= phosphate-buffered saline; *PSMA*= prostate-specific membrane antigen; *tPDT*= targeted photodynamic therapy.

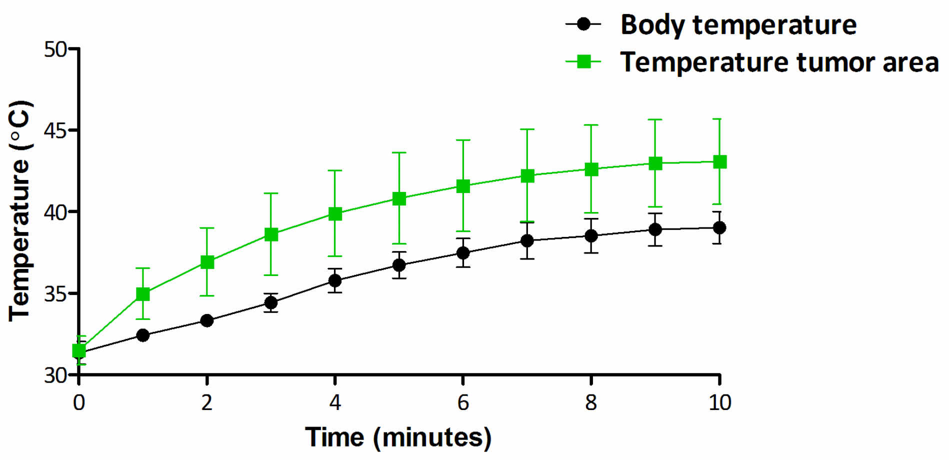

Figure S5. Temperature increase of the mice during PSMA-tPDT. Increasing temperature during irradiation with NIR. The temperature of both the body and the tumor area was monitored during irradiation with 150 J/cm^2 NIR light on the subcutaneous LS174T-PSMA tumors in male BALB/c mice. The graph depicted in black shows the increasing body temperature (31.4 ± 0.7 °C increased to 39.0 ± 1.0 °C). The graph depicted in green shows the increasing temperature of the tumor area (31.5 ± 0.9 °C increased to 43.1 ± 2.6 °C) (n=4). *NIR*= near infrared; *PSMA*= prostate-specific membrane antigen; *tPDT*= targeted photodynamic therapy.

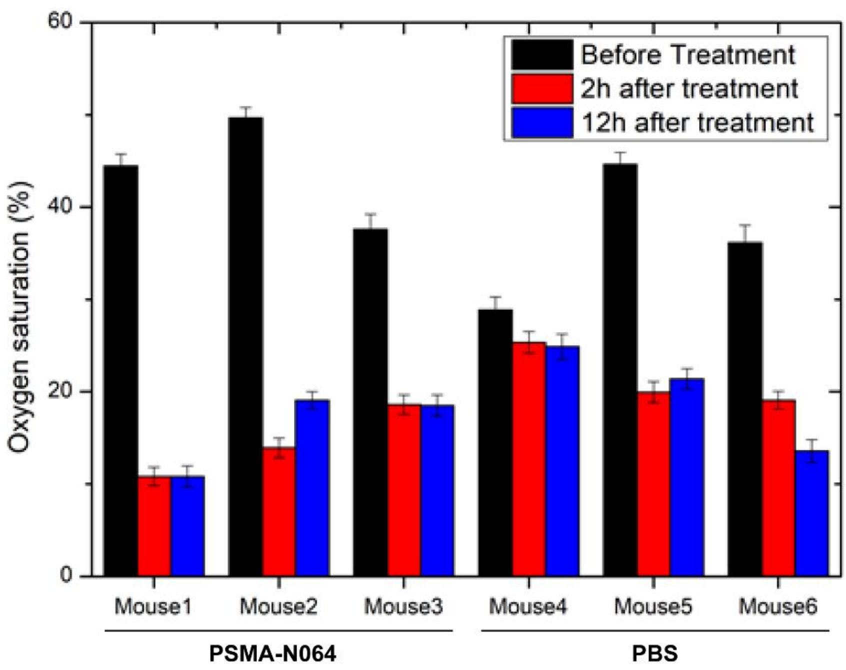

Figure S6. Oxygen saturation before and after PSMA-tPDT measured with photoacoustic imaging. Percentage oxygen saturation was determined in the tumor of the mice before, 2 hrs and 12 hrs after PSMA-tPDT. Treated mice (mouse 1-3) received 3 nmol of PSMA ligand 2 hrs before tPDT with 150 J/cm^2 NIR irradiation. Control mice received PBS (mouse 4-6).
NIR= near infrared; *PBS*= phosphate-buffered saline; *PSMA*= prostate-specific membrane antigen; *tPDT*= targeted photodynamic therapy.

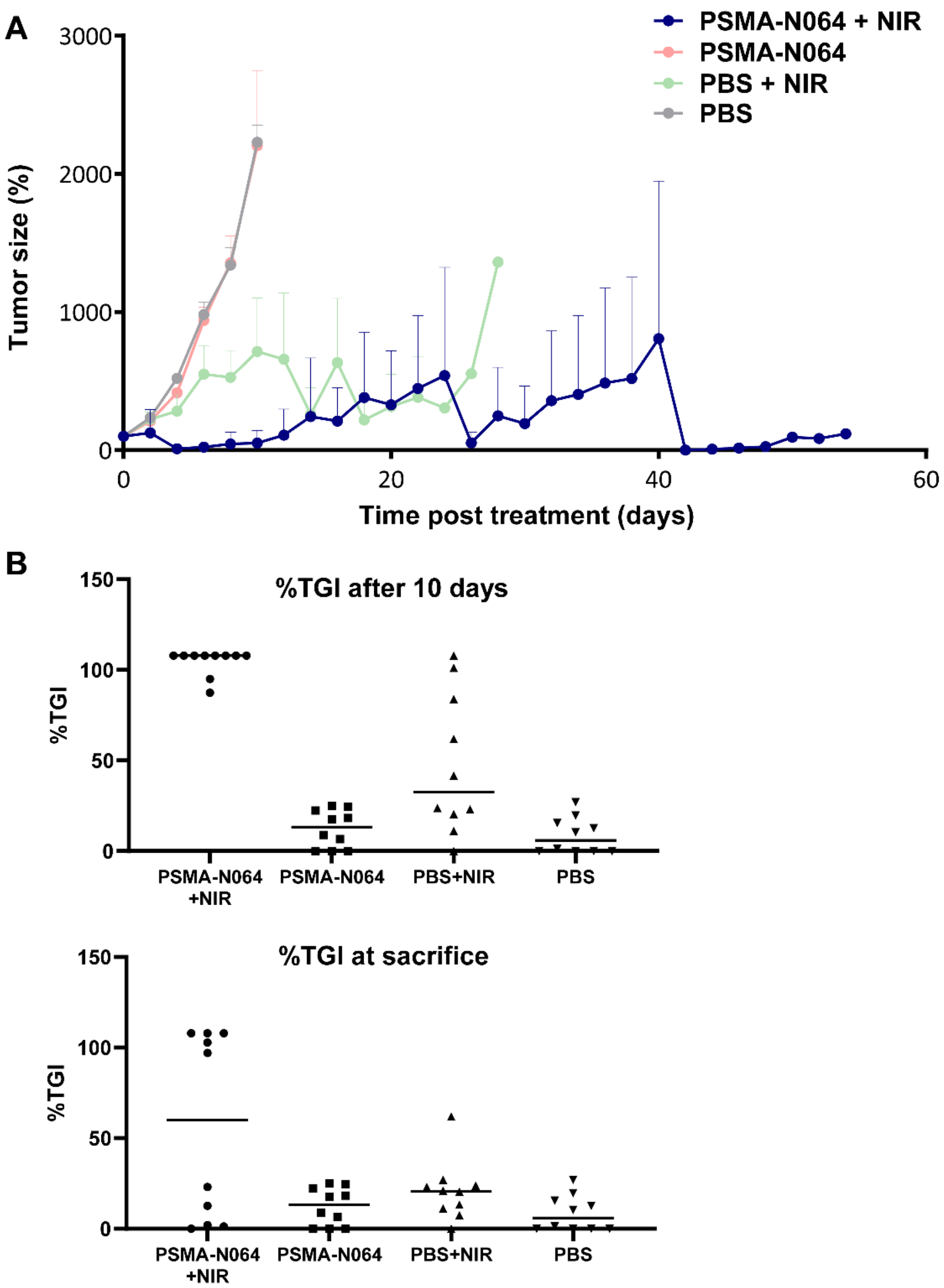

Figure S7. Relative tumor growth and tumor growth inhibition (%TGI) upon PSMA-tPDT. (A) Average relative tumor size of the four treatment groups. **(B)** Percentage tumor growth inhibition for individual mice 10 days after PDT and at day of sacrifice. %TGI = (average tumor size in mm PBS control –tumor size individual treated mouse)/(average tumor size in mm PBS control – average tumor size PBS control initially)*100.

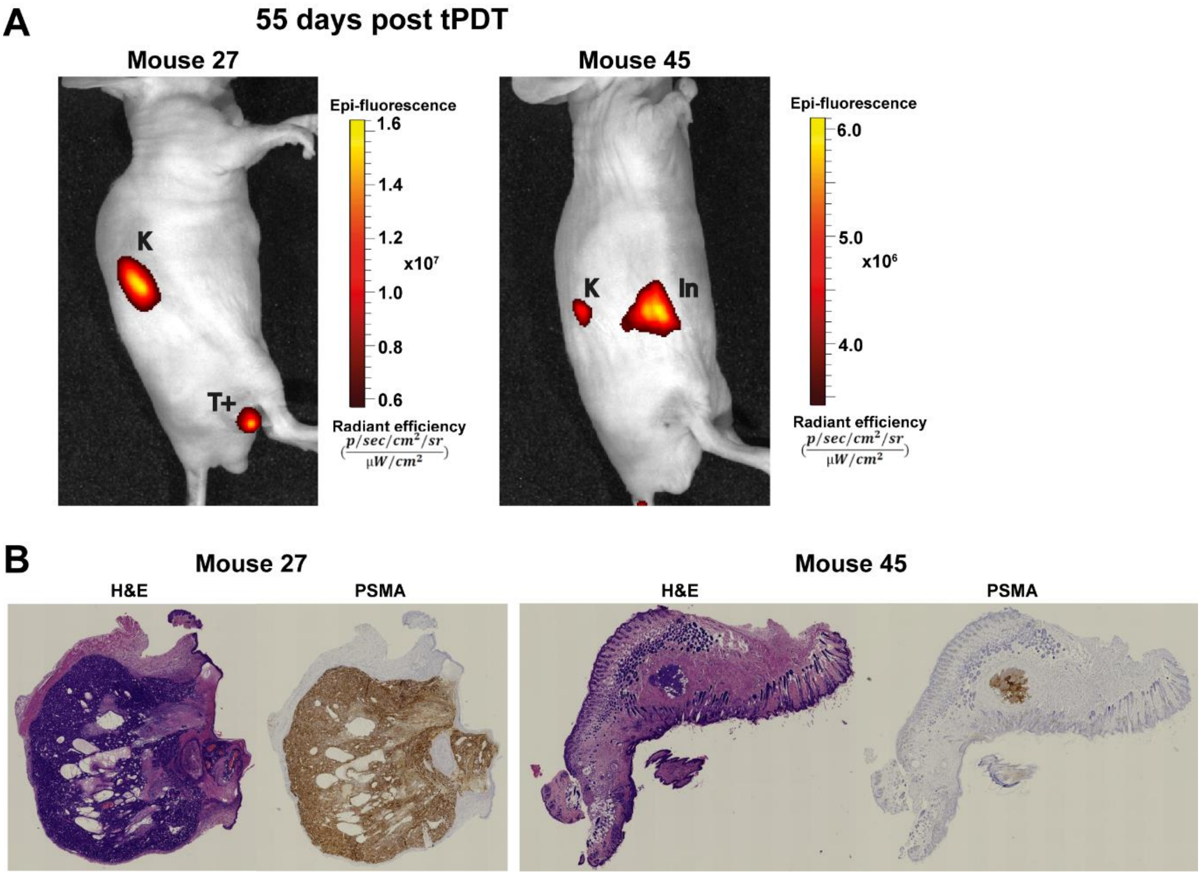

Figure S8. Tumor recurrence 55 days post tPDT in two remaining mice at the end of the experiment.
(A) NIRF images of two remaining mice (group 1) 55 days post tPDT, of which one showed a small recurrence as visualized with NIRF imaging (Mouse 27). T+ = s.c. LS174T-PSMA tumor, K = Kidney and In = intestine. **(B)** PSMA-based immunohistochemical assessment of the tumor site revealed the presence of a small tumor (Mouse 27) and a tiny tumor nodule (Mouse 45) in the two remaining mice at the end of the experiment.
NIRF= near infrared fluorescence; *PSMA*= prostate-specific membrane antigen; *tPDT*= targeted photodynamic therapy.

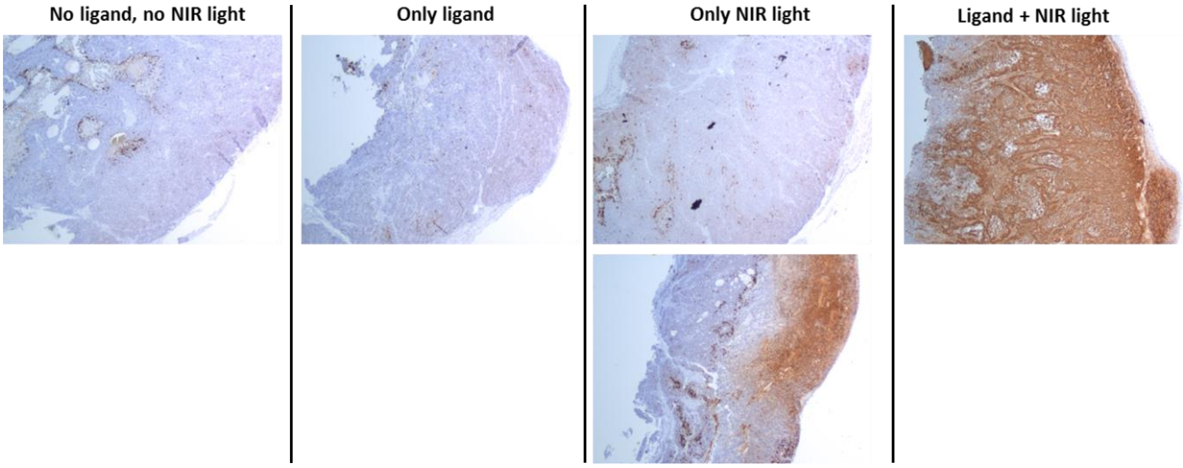

Figure S9. Cleaved caspase-3 staining in tPDT treated mice.
Cleaved caspase-3 staining of subcutaneous LS174T-PSMA tumors after intravenous injection of PBS or 3 nmol of PSMA-N064, followed by NIR light exposure of 150 J/cm^2 (300 mW/cm^2) 2 hrs after injection or no exposure. Tumors were dissected 24 hrs post tPDT.
NIR= near infrared; *tPDT*= targeted photodynamic therapy.
